# Washing our hands of the congenital cytomegalovirus disease epidemic

**DOI:** 10.1186/1471-2458-5-70

**Published:** 2005-06-20

**Authors:** Michael J Cannon, Katherine Finn Davis

**Affiliations:** 1 National Center for Infectious Diseases, Centers for Disease Control and Prevention, Atlanta, Georgia; 2 Rollins School of Public Health, Emory University, Atlanta, Georgia, USA; 3 Nell Hodgson Woodruff School of Nursing, Emory University, Atlanta, Georgia, USA

## Abstract

**Background:**

Each year in the United States, an estimated 40,000 children are born with congenital cytomegalovirus (CMV) infection, causing an estimated 400 deaths and leaving approximately 8000 children with permanent disabilities such as hearing or vision loss, or mental retardation. More children are affected by serious CMV-related disabilities than by several better-known childhood maladies, including Down syndrome, fetal alcohol syndrome, and spina bifida.

**Discussion:**

Congenital CMV is a prime target for prevention not only because of its substantial disease burden but also because the biology and epidemiology of CMV suggest that there are ways to reduce viral transmission. Because exposure to the saliva or urine of young children is a major cause of CMV infection among pregnant women, it is likely that good personal hygiene, especially hand-washing, can reduce the risk of CMV acquisition. Experts agree that such measures are likely to be efficacious (*i.e*., they will work if consistently followed) and the American College of Obstetricians and Gynecologists recommends that physicians counsel pregnant women about preventing CMV acquisition through careful attention to hygiene. However, because of concerns about effectiveness (*i.e*., Will women consistently follow hygienic practices as the result of interventions?), the medical and public health communities appear reluctant to embrace primary CMV prevention via improved hygienic practices, and educational interventions are rare. Current data on the effectiveness of such measures in preventing CMV infection are promising, but limited. There is strong evidence, however, that educational interventions can prevent other infectious diseases with similar transmission modes, suggesting that effective interventions can also be found for CMV. Until a CMV vaccine becomes available, effective educational interventions are needed to inform women about congenital CMV prevention.

**Summary:**

Perhaps no single cause of birth defects and developmental disabilities in the United States currently provides greater opportunity for improved outcomes in more children than congenital CMV. Given the present state of knowledge, women deserve to be informed about how they can reduce their risk of CMV infection during pregnancy, and trials are needed to identify effective educational interventions.

## Background

The history of public health in twentieth century America is replete with successes in the prevention of birth defects and childhood disabilities. Vaccines have virtually eliminated polio, congenital rubella syndrome, and *Haemophilus influenzae *meningitis [1-3]. Educational efforts aimed at preventing fetal alcohol syndrome have reduced maternal alcohol consumption during pregnancy [4]. Prenatal vitamins and folic acid fortification of cereals have lowered rates of neural tube defects [5], while antiretroviral treatments have caused the occurrence of mother-to-child human immunodeficiency virus (HIV) transmission to plummet [6]. Notably absent from the list of successes, however, is the prevention of congenital cytomegalovirus (CMV) disease [7].

**Figure 1:**
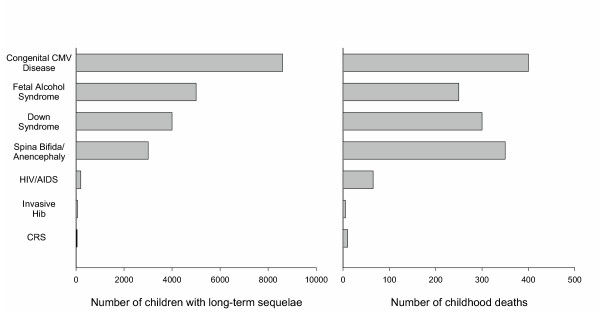
Estimates of the annual burden of prominent childhood diseases and syndromes in the US [3, 5, 6, 8, 51–57]. Assumes 4 million live births per year and 20 million children <5 years of age. Childhood deaths were defined as those occurring <1 year after birth except for Haemophilus influenzae type B (Hib) (<5 years) and HIV/AIDS (<13 years). Where applicable, numbers represent means of published estimates. All estimates should be considered useful for rough comparisons only since surveillance methodology and diagnostic accuracy varied over different studies. CRS, congenital rubella syndrome.

Perhaps no single cause of birth defects and developmental disabilities in the United States currently provides greater opportunity for improved outcomes in more children than congenital CMV. Each year in the United States, an estimated 40,000 children are born with congenital CMV infection, causing an estimated 400 deaths and leaving approximately 8000 children with permanent disabilities such as hearing or vision loss, or mental retardation [8]. The direct annual economic costs of caring for these children are estimated at $1-$2 billion [8,9]. More children are adversely affected by congenital CMV disease than by several better-known childhood diseases or syndromes (Figure 1). Congenital CMV is a prime target for prevention not only because of its substantial disease burden but also because the biology and epidemiology of CMV suggest that there are ways to reduce viral transmission. Unfortunately, by missing prevention opportunities, we in the medical and public health communities are washing our hands of the congenital CMV disease epidemic.

### CMV and congenital CMV disease

As with other human herpesviruses, initial infection by CMV (also known as primary infection) is followed by the establishment of lifelong latent infection, from which periodic reactivation is common [10,11]. Symptoms are usually absent during primary infection and reactivation, but CMV is shed in various bodily secretions, particularly urine and saliva [12]. CMV excretion can be continuous or intermittent, generally lasting several weeks in adults but often continuing for months or years in young children [13-15]. CMV infection is widespread, with estimates of CMV seroprevalence in the United States ranging from 40% to 80% [16-18].

CMV is transmitted person-to-person via close non-sexual contact, sexual activity, breastfeeding, blood transfusions, and organ transplantation [12]. CMV has not been shown to be transmitted via respiratory secretions or aerosolized virus. For the pregnant woman, the most likely source of infection may be contact with the urine or saliva of young children, especially her own children [19,20].

**Figure 2:**
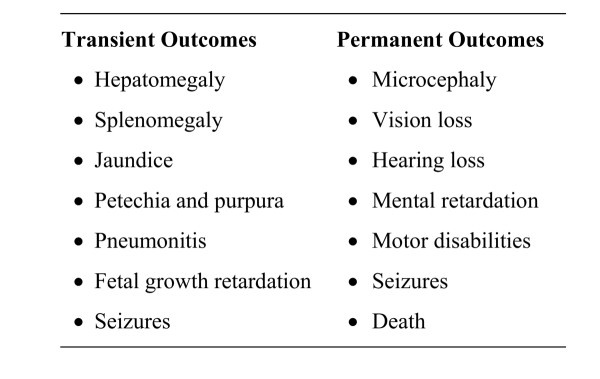
Transient and permanent outcomes among children with congenital CMV disease.

Congenital CMV disease is most likely to occur following a primary infection in the mother. Primary infections occur in 1%-4% of seronegative, pregnant women and lead to fetal infection in 40%-50% of these pregnancies. Maternal CMV reactivation or reinfection with a different CMV strain leads to fetal infection in about 1% of seropositive, pregnant women. Approximately 10% of congenitally infected infants are symptomatic at birth, and of the 90% who are asymptomatic, 10%-15% will develop symptoms over months or even years (Figure 2) [21]. Permanent sequelae can result from CMV infection of the fetus during any trimester, but infection during early fetal development is likely to be especially damaging [22,23]. Since few newborns are screened for CMV, the true impact of congenital CMV infection is underappreciated.

## Discussion

### Reducing the burden of congenital CMV disease

Of all the ways to fight congenital CMV disease, the development of a vaccine is viewed as the most promising. Considerable progress has been made over the last 30 years, but insufficient interest by vaccine manufacturers (Stanley Plotkin, personal communication) and technical challenges make it is uncertain when a vaccine will become available [24]. Avenues for improving outcomes in congenitally infected children have also been explored, including anti-CMV therapies (e.g., ganciclovir) for seriously infected infants [25,26] and supportive care, such as hearing screening, language therapy, and special education [27,28]. In contrast, insufficient emphasis has been given to preventing CMV infection in pregnant women. While women may be infected via several routes, the remainder of this article focuses on preventing transmission via the important child-to-mother route, by encouraging hygienic practices such as frequent hand washing.

**Figure 3:**
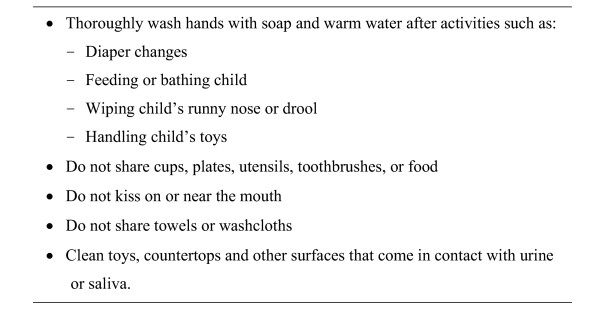
Hygienic practices to reduce risk of CMV infection for women who are pregnant or planning to become pregnant. When interacting with young children, women should assume the children are secreting CMV in their urine and saliva.

A number of experts have suggested that women be educated about hygienic practices for preventing CMV transmission from young children, and there is little dispute over what the prevention guidelines should entail (Figure 3) [7,11,29-32]. This consensus is reflected in current American College of Obstetricians and Gynecologists guidelines, which recommend that physicians counsel pregnant women about preventing CMV acquisition through careful attention to hygiene [33]. Nevertheless, hygienic practices do not appear to be widely discussed by healthcare providers and prospective mothers are often unaware of both CMV disease and the potential benefits of hygienic practices. The virtual absence of a prevention message has been due, in part, to the low profile of congenital CMV. Infection is usually asymptomatic in both mother and infant, and when symptoms do occur, they are non-specific, so most CMV infections go undiagnosed. The prevention message has also been hindered by a sense that infection is unavoidable. For example, a number of authors have urged prevention education for women on the one hand but on the other hand, they have noted that "CMV is neither preventable nor treatable..."[34],"...it is not certain that infections in pregnant women can be prevented by avoiding exposure" [35], "...it is doubtful whether parents will comply with these [behavioral measures in nonstudy settings..." [36], "...there is very little evidence for the efficacy of these strategies and even less for their practical implementation...", and "The only effective prevention strategy relies upon the development of a vaccine." [37] Given the relative invisibility of CMV disease and these mixed messages about prevention education, it is not surprising that healthcare providers do not discuss CMV with their patients and that women are unaware of the risks of CMV infection.

### Preventing CMV infection through hygienic practices

Why the ambivalence toward hygienic practices? Studies have shown that transmission of CMV via the urine and saliva of children is a major cause of infection among pregnant women [19,20]. In addition, more than 100 years of evidence conclusively demonstrates that hand washing reduces risk of infection for a wide range of pathogens [38]. Thus, nearly everyone would agree that, in theory, hand washing can prevent CMV infection because hands are an important vehicle for transmission. The concern, then, is not the *efficacy *of hygienic practices (i.e., Will they work if consistently followed?) but, instead, the *effectiveness *of interventions to promote them (i.e., Will women consistently follow hygienic practices as the result of interventions?).

It is important to recognize the implications of the consensus that hygienic practices are efficacious for preventing CMV transmission. Individual women have the right to know that, under ideal conditions, risk of child-to-mother CMV transmission can be reduced by proper hygienic practices. This is equivalent to the ethical obligation to inform individuals that, under ideal conditions, safer sexual practices will reduce the risk of acquiring HIV. This obligation is independent of whether any particular educational program or intervention is effective. All women of childbearing age, whether they are CMV seropositive or seronegative, carry some risk of new CMV infection during pregnancy and thus should be informed of hygienic practices that reduce that risk. As Revello and Gerna aptly remind us, "...withholding information on possible medical interventions is unethical (and legally risky)" [39]. The terrible burden of congenital CMV disease (Figure 1) should make the provision of such information a priority.

As there is consensus on the efficacy of hygienic practices in preventing CMV transmission, the next step is to evaluate the effectiveness of educational interventions in preventing CMV transmission. Current evidence of effectiveness is promising, but limited. In one study, after non-pregnant women were educated about CMV prevention, hygienic practices improved [40]. In a small study of Houston families, Demmler and colleagues found that behavioral changes prevented transmission of CMV (unpublished report described in Yow and Demmler [7]). Adler and colleagues studied the effectiveness of hygienic practices in a randomized, controlled trial of 39 seronegative, non-pregnant women with young children who were shedding CMV [31]. Although the study was underpowered to detect significant differences in infection rates between the intervention and control groups (and thus the intervention was deemed unsuccessful by some), seroconversion rates decreased as CMV education and support increased. Furthermore, in the same study, 14 pregnant women were educated regarding hygienic practices and then followed for comparison with the randomized groups; none of the women seroconverted – a significant difference compared with the randomized groups. A more recent study also reported that pregnant women who received an intervention involving hygienic practices were significantly less likely to acquire CMV infection than were non-pregnant women [41].

More conclusive evidence of effectiveness can be found in the literature on community-based interventions for the prevention of other infectious diseases with similar transmission modes. For example, a meta-analysis found that community intervention trials that encouraged washing hands with soap reduced the risk of diarrheal diseases by 47% [42]. Hand-washing programs reduced respiratory illness among military recruits [43] and children in daycare [44], and interventions involving hand sanitizers reduced absenteeism among elementary school teachers and children [45]. An in-depth review of the literature would be useful for determining the key factors associated with the success of these and other community interventions.

Although all women of childbearing age deserve to be informed about CMV, interventions for preventing CMV transmission are most likely to be effective for pregnant women, who tend to be highly motivated, often changing behavior to protect the health of their developing fetuses. As a case in point, 25% of low-income smokers spontaneously quit smoking during pregnancy [46]; this percentage is higher than that achieved by most smoking cessation programs [47]. Studies by Adler and colleagues suggest that motivation for avoiding CMV infection is considerably higher among pregnant than non-pregnant women [31,41].

In sum, the evidence to date gives every indication that effective interventions can be found for preventing CMV infection among pregnant women. Thus, the paradigm must shift from wondering whether such interventions will be effective to developing and evaluating interventions until effective ones are identified.

### Next steps

Given the consequences of allowing the congenital CMV disease epidemic to continue unabated, it is imperative that women receive the educational message about congenital CMV disease prevention (see ) [48]. Promotion of this message could translate years of careful CMV research into an immediate public health benefit. Encouraging hygienic practices would be relatively inexpensive (requires no laboratory testing or additional doctor visits), ethically responsible (allows women to make informed decisions), and likely to prevent disabilities and save lives. Based on studies of the economic impact of future CMV vaccines, which estimate savings of $50,000 per quality adjusted life-year saved [8], it is likely that CMV education efforts would provide a highly favorable cost-benefit ratio as well. CMV educational messages should emphasize hygienic practices as a precaution for all women who are pregnant or planning to become pregnant, and reasonable but not extreme measures for minimizing risk during interactions with young children (Figure 3). In many instances, a CMV education message could build upon and strengthen other public health messages about infection prevention through improved hand hygiene. Once effective hand-hygiene messages are identified, more ambitious goals might also be considered, such as prevention of sexual transmission or transmission between children in daycare.

Enlisting the support of healthcare providers to convey the gravity of CMV infection and the importance of good hygienic practices is crucial. Healthcare providers have many opportunities to provide women with such information, such as during annual gynecological exams or well-baby visits as women accompany their children to the pediatrician's office. Other steps, such as community education by healthcare providers (including information sessions with daycare directors, daycare providers, and parents) and provision of information by the Public Health Service and other professional organizations, can supplement the healthcare provider's information [49]. Success in delivering the message will depend on the active involvement of all relevant healthcare professionals.

In addition, trials are needed to identify effective community-based interventions for preventing CMV transmission to pregnant women. These trials will be important for quantifying the effectiveness of the proposed hygienic practices and for assessing the proportion of CMV infections that result from child-to-mother transmission as opposed to other routes, such as sexual transmission. However, such trials should not delay nor hinder the educational effort, just as we would not wait to inform skydivers about the prudence of parachute use because the results of controlled trials are not yet in. "As with many interventions intended to prevent ill health, the effectiveness of parachutes has not been subjected to rigorous evaluation by using randomised controlled trials...*[One option] is that we accept that, under exceptional circumstances, common sense might be applied when considering the potential risks and benefits of interventions *[our emphasis]."[50] Common sense tells us that avoiding CMV-laden secretions will prevent transmission and that the potential benefits of educational interventions far outweigh the potential risks.

Although an effective CMV vaccine would be ideal and vaccine development deserves increased support, hope for a vaccine tomorrow should not stand in the way of a vigorous educational message today. Prevention through improved hygienic practices will not be easy, but washing our hands of the problem by staying the present course guarantees that each year in the United States, hundreds of children will die and thousands of others will swell the ranks of CMV-affected children growing up with serious disabilities. Just as the medical and public health communities are successfully meeting the challenges posed by polio, rubella, HIV, and neural tube defects, now is the time to meet the congenital CMV challenge head on by making awareness and prevention high priorities.

## Summary

• Each year in the United States, congenital CMV

- causes an estimated 400 deaths

- leaves more than 8000 children with permanent disabilities such as hearing or vision loss, or mental retardation

• Exposure to the saliva or urine of young children is a major cause of CMV infection among pregnant women.

• Risk of CMV infection is likely to be reduced by careful attention to good personal hygiene, especially hand-washing.

• Women should be informed about how to reduce their risk of CMV infection during pregnancy.

• Trials are needed to identify educational interventions that are effective in preventing CMV infection.

## Competing interests

The author(s) declare that they have no competing interests.

## Authors' contributions

MJC and KFD contributed equally to the drafting and critical revision of the manuscript. Both authors read and approved the final manuscript.

## Pre-publication history

The pre-publication history for this paper can be accessed here:


